# The evolution of diagnosis from symptom onset to death in progressive supranuclear palsy (PSP) and corticobasal degeneration (CBD) compared to Parkinson’s disease (PD)

**DOI:** 10.1007/s00415-023-11629-x

**Published:** 2023-03-27

**Authors:** Diane M. A. Swallow, Carl E. Counsell

**Affiliations:** Institute of Applied Health Sciences, University of Aberdeen, Polwarth Building, Foresterhill, Aberdeen, AB25 2ZD UK

**Keywords:** Progressive supranuclear palsy, Corticobasal degeneration, Parkinson’s disease, Misdiagnosis, Delayed diagnosis, Survival

## Abstract

**Background:**

Misdiagnosis and delayed diagnosis in progressive supranuclear palsy (PSP) and corticobasal degeneration (CBD) are common. Few studies have systematically evaluated the diagnostic process from symptom onset to death in representative cohorts.

**Methods:**

All PSP/CBD cases (*n* = 28/2) and age-sex matched Parkinson’s disease (PD) cases (*n* = 30) were identified from a UK prospective incident Parkinsonism cohort. Medical and research records were reviewed to compare median times from first index symptom to key diagnostic milestones and the nature/timing of secondary care referral and review.

**Results:**

Index symptoms were similar apart from more tremor in PD (*p* < 0.001) and more impaired balance (*p* = 0.008) and falls (*p* = 0.004) in PSP/CBD. PD was diagnosed a median 0.96 years after index symptom. In PSP/CBD the median times from index symptom to identifying parkinsonism and then including PSP/CBD in the differential diagnosis and the final diagnosis were 1.88, 3.41 and 4.03 years, respectively (all *p* < 0.001). Survival from symptom onset in PSP/CBD and PD was not significantly different (5.98 vs 6.85 years, *p* = 0.72). More diagnoses (*p* < 0.001) were considered in PSP/CBD. Prior to diagnosis, PSP/CBD patients had more recurrent emergency attendances (33.3% vs 10.0%, *p* = 0.01) and were referred to more specialities than PD (median 5 vs 2). Time to any outpatient referral (0.70 vs 0.03 years, *p* = 0.025) and to specialist movement disorder review (1.96 vs 0.57 years, *p* = 0.002) was longer in PSP/CBD.

**Conclusions:**

The duration and complexity of the diagnostic journey were greater in PSP/CBD than age-sex matched PD but can be improved. In this older cohort, there was little difference in survival from symptom onset in PSP/CBD and age-sex matched PD.

**Supplementary Information:**

The online version contains supplementary material available at 10.1007/s00415-023-11629-x.

## Introduction

Progressive supranuclear palsy (PSP) and corticobasal degeneration (CBD) are rare, neurodegenerative tauopathies. Clinicopathological studies highlight the extent of misdiagnosis in PSP and CBD [1–4], but are susceptible to selection bias, favouring those with atypical features or those diagnosed in specialist, research active centres. They also primarily compare pathological diagnoses with ante-mortem clinical diagnoses at first and final clinical presentation [2, 5], leaving substantial gaps in our understanding of the evolution of the diagnostic process. Few studies have systematically described the diagnostic pathway in PSP/CBD from diagnosis to death in representative cohorts to provide a greater understanding of the occurrence of diagnostic errors and identify opportunities to improve diagnostic timeliness and accuracy. This is important for clinical care and to ensure individuals with PSP/CBD can be identified early in their disease course while they still meet eligibility criteria for disease-modifying clinical trials. We therefore compared the diagnostic process from symptom onset to death in PSP/CBD and age-sex matched PD cases recruited to a prospective population-based incident cohort of parkinsonism in the UK [6].

## Methods

The PINE study is an incident cohort of PD and other forms of parkinsonism with prospective life-long annual follow-up in the North-East of Scotland [6]. Multiple, overlapping methods of case ascertainment identified all patients with previously undiagnosed degenerative or vascular parkinsonism during two incidence periods (2002–2004 and 2006–2009). All potential incident cases were invited to undergo clinical assessment by a neurologist with a specialist interest in movement disorders or by a supervised trainee (baseline assessment). Patients were included as incident cases if they had parkinsonism defined by two or more of four cardinal motor features (rest tremor, bradykinesia, rigidity, and postural instability), and the date of first diagnostic suspicion of parkinsonism by a GP or hospital doctor (whoever first considered it) occurred within the incident period. Patients consented both to life-long annual clinical follow-up (outcome measures previously described in detail [6]) and primary/secondary care medical record review (enabling identification of symptoms/signs pre-study entry).

All incident cases with a final clinical (or post-mortem) diagnosis of PSP or CBD in the PINE study were included in the study sample. Although the term CBD is increasingly restricted to pathologically confirmed cases due to the diversity of clinical presentations in those with CBD pathology, with the term corticobasal syndrome (CBS) preferred, the term CBD is used to maintain consistency with previous publications in the PINE cohort. PSP/CBD cases were age- and sex-matched to incident PD cases randomly selected from all recruited PD cases.

At baseline and at subsequent reviews, the specific cause of the parkinsonian syndrome was made based on history, standardised examination (including features of an atypical parkinsonian syndrome such as eye movements, dystonia, myoclonus, apraxia, ataxia), response to dopamine replacement treatment and available imaging findings guided by specific diagnostic criteria available at the time (in PSP the 1996 consensus criteria [7], in CBD Lang’s criteria [8] and in PD the UK PD Brain Bank Criteria [9]). All patients were invited to consent to a post-mortem examination. Final diagnoses in the current analysis are based on an individual’s final clinical diagnosis following review of all clinical and imaging data after death or pathological diagnosis.

Baseline (time of first assessment and entry to PINE study) case demographic and clinical characteristics were extracted. The full primary care, secondary care and research record for each case was systematically hand-searched retrospectively to identify relevant data. The onset of the diagnostic process was defined as the date of index symptom onset. Index symptom was defined as the first documented (not recalled) symptom, in the primary or secondary care record, which persisted throughout the course of illness (to maximise specificity of symptoms relating to their parkinsonian diagnosis). A list of motor, non-motor and neuropsychiatric symptoms and signs systematically sought from the records are detailed in Appendix 1. At their baseline visit patients also recalled the onset of their first parkinsonian symptom, defined as patient recalled index symptom.

A documented primary diagnosis was a clinician’s single best explanation for the patient’s clinical presentation, while differential diagnoses were documented alternative explanations for the patient’s presentation. Prior to PINE study referral clinicians were general practitioners (GPs) or hospital specialists (any), while after study entry patients had at least annual review by a movement disorder specialist in addition to other hospital specialists. Both syndromic and disease-specific diagnoses, mixed diagnoses, and exogenous factors such as alcohol or an infection (if a clinician’s single best explanation for symptoms) were considered discrete diagnoses. The date each primary diagnosis was first proposed or re-diagnosed if subsequent diagnostic revisions were made (to determine the frequency of changing diagnoses) was collated. If a specific diagnosis of PSP/CBD or PD was only made at post-mortem or following case note review after death, the date of death was used as the date of diagnosis.

Secondary care referrals and attendances after index symptom onset were reviewed chronologically. Emergency attendances were defined as both unscheduled Accident and Emergency (A&E) assessments with subsequent discharge or those requiring hospital admission. Elective outpatient referrals were new referrals made by GPs or other secondary care physicians. Healthcare episodes which were the first documented occurrence of symptoms relevant to PSP/CBD were included. Follow-up secondary care appointments initiated prior to index symptom onset for unrelated co-morbidities were not counted as first secondary care contacts after index symptom onset. Outpatient referrals or elective inpatient episodes unequivocally due to unrelated specific symptoms/signs (for example, a breast lump or chemotherapy initiation), were excluded.

The proportion of patients with each individual symptom and sign at the onset of the diagnostic process was compared between PSP/CBD and PD using the Chi-square test. Using the dates associated with specific diagnoses, the average diagnostic time intervals (median, interquartile range [IQR]) from index symptom to key diagnostic milestones including diagnosis of a parkinsonian syndrome, inclusion of a correct diagnosis of PSP/CBD or PD in differential diagnoses, PSP/CBD or PD as the final primary clinical diagnosis, and death, were calculated and compared between groups using the Mann–Whitney test. The average number (median, IQR) of primary diagnoses, differential diagnoses, and the number of chronological changes to a patient’s primary diagnosis from index symptom to death, and both before and after movement disorder specialist input (PINE study team review), were compared using the Mann–Whitney test.

The mode of entry to secondary care, including the number of emergency attendances and elective referrals, subdivided by speciality, was compared between groups using the Chi square test. The timing, reason for attendance, outcome and duration of admission of the first unscheduled emergency contact in PSP/CBD and age-sex matched PD was compared using information from individually extracted episodes. For elective outpatient referrals, the documented specialty, referrer diagnosis and the primary diagnosis after each secondary care healthcare contact was also summarised. Time intervals (median, IQR) from index symptom onset to the first outpatient secondary care referral and first movement disorder review were compared using Mann–Whitney test.

## Results

Of 315 recruited to the PINE cohort with confirmed parkinsonism on follow-up, clinically diagnosed causes of parkinsonism at baseline included PD (*n* = 200, 63.6%), DLB (*n* = 37, 11.7%), vascular parkinsonism (*n* = 33, 10.5%), PSP/CBD (*n* = 30, 9.5%), MSA (*n* = 11, 3.5%) and dementia with associated parkinsonism (*n* = 4, 1.3%).

All incident cases, PSP (*n* = 28), CBD (*n* = 2) and PD (*n* = 30), were deceased at the time of data extraction (February 2017). PSP and CBD were combined due to the small numbers of cases with CBD. All clinically diagnosed cases with PSP who came to post-mortem had PSP (*n* = 4, 14.3%) while in five (17.9%) PSP cases, the correct diagnosis was only identified at post-mortem. The clinical diagnoses of nine (30.0%) sampled PD cases that underwent post-mortem were also all confirmed pathologically (of 56 clinically diagnosed cases in the entire PINE cohort who underwent post-mortem, PD has been confirmed in 49 [87.5%] of cases). At baseline assessment, there were no statistically significant differences between PSP/CBD and PD with respect to age, sex, ethnicity, marital status, education, socio-economic status or medical comorbidities (Table 1). Motor severity (UPDRS 3) was higher in the PSP/CBD group while there was weak evidence of a significant difference in the total cognitive scores (MMSE) between groups.

**Table 1:** Baseline demographic and clinical characteristics of 30 PSP and CBD and 30 age-sex matched PD controls

	PSP/CBD *n* = 30	PD *n* = 30	*p*-Value*
Age at index symptom (years), mean (SD)	75.5 (9.4)	74.6 (13.3)	0.770
Age at baseline (years), mean (SD)	77.9 (9.1)	77.5 (8.5)	0.741
Sex, female	15 (50.0)	15 (50.0)	1.000
Ethnicity, white	30 (100)	30 (100)	1.000
Marital status			
Married	16 (53.3)	14 (46.7)	0.606
Single/widowed/divorced	14 (46.7)	16 (53.3)	
Education			
Secondary	22 (73.3)	26 (86.7)	0.255
Tertiary	6 (20.0)	4 (13.3)	
Missing	2 (6.7)	0 (0.0)	
Socio-economic status			
Depcat 1–3 (most affluent)	18 (60.0)	17 (56.7)	0.793
Depcat 4–7 (least affluent)	12 (40.0)	13 (43.3)	
Duration of follow-up from baseline to final visit (years), mean (SD)	3.2 (2.6)	4.7 (2.7)	**0.030**
UPDRS (mean, SD)			
Part 1	2.7 (2.3)	2.1 (1.9)	0.3638
Part 2	16.5 (6.9)	9.8 (5.2)	** < 0.001**
Part 3	36.9 (18.1)	25.9 (10.2)	**0.006**
Part 4	0.07 (0.37)	0.07 (0.26)	0.9550
MMSE (mean, SD)	26.3 (3.8)	28.3 (1.1)	0.059
Hoehn & Yahr			
0–2.5	12 (40.0)	22 (73.3)	**0.003**
3	4 (13.3)	4 (14.3)	
> 3	14 (46.7)	2 (6.7)	
Schwab and England Score			
< 80	19 (63.3)	6 (20.7)	** < 0.001**
≥ 80	11 (36.7)	23 (79.3)	
Charlson Comorbidity Index			
0	10 (33.3)	11 (36.7)	0.951
1	10 (33.3)	10 (33.3)	
> 1	10 (33.3)	9 (30.0)	

Bold values in column which are statistically significant

*CBD* corticobasal degeneration; *Depcat* small postcode measure of socioeconomic status based on proportions of overcrowding, male unemployment, low occupational social class and car ownership; *MMSE* mini-mental state examination; *PD* Parkinson’s disease; *PSP* progressive supranuclear palsy; *SD* standard deviation; *UPDRS* unified Parkinson’s disease rating scale

Data are number (percentage) unless stated otherwise

^*^Two sample *t*-test or Chi-square test

Both PSP/CBD and PD patients experienced a range of index symptoms (Table 2), with most (*n* = 20, 66.7% in both groups) first identified in primary care. The average age at index symptom onset was 75.5 years (SD 9.4) in PSP/CBD and 74.6 years (SD 13.3) in PD. Only tremor in PD patients (60% PD, 3.3% PSP/CBD, *p* < 0.001), and impaired balance (23.3% PD, 56.7% PSP/CBD, *p* = 0.008) and falls (10.0% PD, 43.3% PSP/CBD, *p* = 0.004) in PSP/CBD patients, were significantly different between groups at symptom onset, consistent with differences in index signs where, in addition to the above, rigidity was also more prevalent in PD (43.3% PD, 16.7% PSP/CBD, *p* = 0.024) (Supplementary Table 1). The overall difference between patient recalled and documented index symptom onset (patient reported minus documented symptoms) was 0.00 (IQR − 0.72 to 2.49) years in PSP/CBD and − 0.55 (− 1.05 to − 0.01) years in PD (i.e., the date of documented index symptom onset in PD was earlier than patient recalled index symptom).

**Table 2:** Comparison of index symptom frequency (categorised) in PSP/CBD and PD

Index symptoms^a^	PSP/CBD	PD	*p*-Value*
	*n* = 30	*n* = 30	
Tremor	1 [1 PSP] (3.3%)	18 (60.0%)	** < 0.001**
Slowness/gait disturbance	11 [11 PSP] (36.7%)	13 (43.3%)	0.598
Impaired balance	17 [16 PSP, 1 CBD] (56.7%)	7 (23.3%)	**0.008**
Falls	13 [13 PSP] (43.3%)	3 (10.0%)	**0.004**
Speech	3 [3 PSP] (10.0%)	2 (6.7%)	0.640
Neuropsychiatric	2 [2 PSP] (6.7%)	1 (3.3%)	0.554
Cognitive	4 [3 PSP, 1 CBD] (13.3%)	2 (6.7%)	0.389
Incoordination	3 [2 PSP, 1 CBD] (10.0%)	0 (0.0%)	0.076
Sensory	1 [1 CBD] (3.3%)	0 (0.0%)	0.313
Stiffness	1 [1 PSP] (3.3%)	3 (10.0%)	0.301
Pain	0 (0.0%)	1 (3.3%)	0.313
Swallowing	2 [2 PSP] (6.7%)	0 (0.0%)	0.150
Fatigue	1 [1 PSP] (3.3%)	1 (3.3%)	1.000
Sleep disturbance, including RBD	0 (0.0%)	1 (3.3%)	0.313
Urinary	1 [1 PSP] (3.3%)	0 (0.0%)	0.313

Bold values in column which are statistically significant

*PSP* progressive supranuclear palsy; *CBD* corticobasal degeneration; *PD* Parkinson’s disease; *RBD* REM sleep behaviour disorder

^*^Chi-squared test

^a^Participants could have multiple symptoms at onset. Neuropsychiatric symptoms included mood disturbance (depression, anxiety) or altered personality/behaviour, cognitive symptoms included symptoms such as memory difficulties or slower processing speed)

All stages of the diagnostic process were significantly longer in PSP/CBD compared to PD (Table 3). Overall, individuals with PD received their final, unchanging diagnosis within 1 year (median 0.96 years) from first documented symptom, while for those with PSP/CBD it took nearly 2 years before identification of the parkinsonian syndrome, a further one-and-a-half years before PSP/CBD were considered amongst possible differential diagnoses and approximately 4 years to a final, unrevised diagnosis. However, the time interval between index symptom to death in PSP/CBD (5.98 years) was not statistically significantly different to that of age-sex matched individuals with PD (6.85 years). Time intervals calculated from patient recalled rather than documented index symptom (Supplementary Table 2), and from index sign (Supplementary Table 3) resulted in slightly different time intervals but with similarly strong evidence of longer time intervals to diagnosis in PSP/CBD.

**Table 3:** Comparison of time intervals from documented index symptom to diagnosis in PSP/CBD and age-sex matched PD

Median (IQR) years from index symptom:	PSP/CBD *n* = 30	PD *n* = 30	*p*-Value*
To diagnosis of a parkinsonian syndrome	1.88 (1.04, 3.89)	0.14 (0.00, 0.77)	** < 0.001**
To specific^a^ diagnosis in differential diagnoses	3.41 (1.71, 5.74)	0.18 (0.00, 1.49)	** < 0.001**
To specific diagnosis as primary clinical diagnosis (initial)	4.03 (2.30, 5.97)	0.37 (0.00, 1.54)	** < 0.001**
To specific diagnosis as primary clinical diagnosis (unchanging)	4.03 (2.31, 6.19)	0.96 (0.08, 4.28)	** < 0.001**
To death	5.98 (3.70, 7.71)	6.85 (3.45, 8.98)	0.723

Bold values in column which are statistically significant

*PSP* progressive supranuclear palsy; *CBD* corticobasal degeneration; *PD* Parkinson’s disease

^*^Mann–Whitney

^a^Specific diagnosis means either PSP, CBD or PD

Over this period (index symptom onset to death), individuals with PSP/CBD were given a median of 4.0 discrete primary diagnoses (both parkinsonian and non-parkinsonian diagnoses) compared with 2.0 primary diagnoses in the PD group (*p* < 0.001) (Table 4). More differential diagnoses were also considered in PSP/CBD (6.0) compared to PD (3.0), the greater diagnostic uncertainty evidenced in more (4.0) ante-mortem chronological changes to the primary diagnosis changes in PSP/CBD compared to 2.0 in PD. Prior to movement disorder review PSP/CBD patients received 3.0 primary diagnoses, compared to 1.0 in PD on average, but there were no significant differences in the frequency of changes to the diagnosis after PINE study entry (i.e., revisions within a specialist movement disorder service).

**Table 4:** The average frequency and changes to primary and differential diagnoses in PSP/CBD and PD from index symptom to death

Median (IQR)	PSP/CBD *n* = 30	PD *n* = 30	*p*-Value*
Primary diagnoses			
Overall total (not including “none”)	4.0 (2.8, 6.0)	2.0 (1.0, 3.0)	** < 0.001**
Total parkinsonian	2.0 (2.0, 3.0)	1.0 (1.0, 2.0)	**0.001**
Total non-parkinsonian	1.5 (0.0, 2.3)	0.0 (0.0, 1.0)	**0.001**
Total prior movement disorder review	3.0 (2.0, 5.0)	1.0 (1.0, 2.0)	** < 0.001**
Differential diagnoses (ante-mortem)			
Overall total	6.0 (4.0, 8.3)	3.0 (2.0, 5.0)	** < 0.001**
Total parkinsonian	4.0 (3.0, 5.0)	2.0 (1.0, 4.0)	**0.001**
Total non-parkinsonian	2.0 (0.0, 3.3)	1.0 (0.0, 1.0)	**0.002**
Chronological revisions to primary diagnosis (ante-mortem)			
Total^a^	4.0 (1.8, 6.0)	2.0 (0.0, 3.3)	**0.001**
Movement disorder revisions only	0.0 (0.0, 1.3)	0.0 (0.0, 1.0)	0.377
All speciality revisions after first movement disorder review	1.0 (0.0, 3.3)	0.0 (0.0, 1.0)	0.106
Episodes no diagnosis (including GP)	1.0 (0.0, 2.0)	0.0 (0.0, 0.8)	**0.021**
Episodes no diagnosis (excluding GP)	0.0 (0.0, 1.0)	0.0 (0.0, 0.0)	0.054

Bold values in column which are statistically significant

Numbers are median (interquartile range)

*PSP* progressive supranuclear palsy; *CBD* corticobasal degeneration; *PD* Parkinson’s disease

^*^Mann–Whitney

^a^Not including healthcare episodes where no diagnosis was made

From index symptom onset, in 33% of individuals with PSP/CBD (*n* = 10, all PSP, mean age 78.2 [SD 8.1], seven women), compared to 10.0% with PD (*n* = 3, all women, mean age 79.7 [SD 6.3]) (*p* = 0.057) their first secondary care contact was an unscheduled healthcare episode. Most emergency presentations in both PSP and PD occurred at, or rapidly after, the date of index symptom onset. In the PSP/CBD subgroup the majority of first emergency presentations were due to falls (*n* = 8), half resulting in injury, and most (*n* = 8) requiring an inpatient stay (median duration 16.0 days). While all three PD emergency presentations also required admission, their admissions were significantly shorter (median 5.0 days, *p* = 0.57). No unscheduled first secondary care contacts resulted in a diagnosis of parkinsonism, PSP/CBD or PD specifically. Unlike PD patients however, for whom their next secondary care contact was a scheduled outpatient review, individuals with PSP/CBD had a higher burden of recurrent emergency attendances (10% PSP/CBD vs 10% PD with one attendance, 10% PSP/CBD vs 0% PD with two to three attendances, and 13.3% PSP/CBD vs 0% PD with four or more attendances) (*p* = 0.011). Of a total of 27 emergency attendances in the PSP/CBD group (Supplementary Table 4), prior to outpatient referral or inpatient diagnosis, over half were due to falls, many resulting in serious fall sequelae. Twenty-four (88.9%) of all PSP/CBD emergency attendances were admitted under Care of the Elderly (COTE) (7 [29.2%]), orthopaedics (5 [20.8%]), psychiatry (5 [20.8%]), general medicine (4 [16.7%]), or A&E (3 [12.5%]).

Of those in whom their first contact with secondary care after symptom onset was in an outpatient setting (*n* = 20, 66.7% PSP/CBD compared to *n* = 27, 90.0% PD), in approximately a third in both the PSP/CBD (*n* = 6) and PD group (*n* = 9), the date the outpatient referral was made was the date of their index symptom/sign. Overall, however, the median time interval from index symptom to primary care referral for elective outpatient review was significantly longer in PSP/CBD (0.70 years, IQR 0.11, 2.86) compared to PD (0.03 years, IQR 0.00, 0.72) (*p* = 0.025). In PD nearly all (*n* = 25 of 27, 92.6%) referrals were sent to neurology or COTE, whereas in PSP/CBD over a third (*n* = 7 of 20 referrals, 35%) of GP referrals were to specialists without movement disorder expertise, including orthopaedics (10.0% PSP/CBD vs 3.7% PD), ear nose and throat (5% PSP/CBD vs 3.7% PD), psychiatry (5% PSP/CBD vs 0% PD), or stroke medicine (10.0% PSP/CBD vs 0% PD) (all *p* > 0.05).

In all 27 PSP/CBD cases in whom an outpatient secondary care referral was eventually made, a specific referrer diagnosis of PSP/CBD was never proposed. Parkinsonism was proposed in 9 (33.3%) referrals, but 8 (29.6%) individuals were referred without a suspected diagnosis and 9 (33.3%) with non-parkinsonian diagnoses. By contrast, in the 30 referred PD cases, PD was proposed by 19 (63.3%) referrers, and parkinsonism in 2 (6.7%) cases. While 9 (30.0%) PD cases were referred without diagnosis, non-parkinsonian diagnoses were never proposed.

Once individuals reached secondary care (all specialties), at the initial outpatient review, parkinsonism was diagnosed in 11 (40.7%) in the PSP/CBD group and 20 (66.7%) in the PD group. A specific correct diagnosis was made in 3 (11.1%) initial outpatient reviews in the PSP/CBD group (all movement disorder specialist reviews), and 18 (60.0%) PD initial outpatient reviews. Unsurprisingly, none of the referrals to specialities other than neurology or COTE resulted in a diagnosis of parkinsonism. Indeed, of all healthcare interactions in PSP/CBD in both primary care and secondary care (*n* = 108), prior to movement disorder specialist review (Table 5), no primary diagnosis was made in many (31.5%). Parkinsonism was identified in 25.9% episodes, but 13.9% episodes resulted in cerebrovascular diagnoses, 17.6% neuropsychiatric diagnoses, 5.6% other neurological diagnoses and 5.6% a variety of other non-neurological diagnoses or non-diagnostic descriptors. In PD, while in a similar number of episodes (30.4%) individuals did not receive any diagnosis, a higher number of episodes (55.4%) led to a diagnosis of parkinsonism.

**Table 5:** Primary diagnoses made in PSP/CBD and age-sex matched PD cases prior to movement disorder review

Healthcare episode	Primary diagnoses PSP/CBD (*n* = 108)	Primary diagnoses PD (*n* = 56)	*p*-Value*
Reviewed with no diagnosis	**34 (31.5)**	**17 (30.4)**	0.883
Parkinsonism	**28 (25.9)**	**31 (55.4)**	** < 0.001**
PD	15 (53.6)	23 (74.2)	
Unspecified parkinsonism	7 (25.0)	3 (9.7)	
VP	2 (7.1)	2 (6.5)	
Drug-induced parkinsonism	1 (3.6)	1 (3.2)	
Atypical parkinsonism	1 (3.6)	0 (0.0)	
Vascular pseudo-parkinsonism	1 (3.6)	0 (0.0)	
DLB	0 (0.0)	2 (6.5)	
CBD	1 (3.6)	0 (0.0)	
Vascular diagnoses	**15 (13.9)**	**1 (1.8)**	**0.012**^a^
TIA/Stroke	11 (73.3)	1 (100.0)	
Vascular disease	4 (26.7)	0 (0.0)	
Neuropsychiatric	**19 (17.6)**	**0 (0.0)**	** < 0.001** ^a^
Alzheimer’s plus	3 (15.8)	0 (0.0)	
FTD	4 (21.1)	0 (0.0)	
Vascular dementia	2 (10.5)	0 (0.0)	
Dementia unspecified	5 (26.3)	0 (0.0)	
Other	5 (26.3)	0 (0.0)	
Other neurological	**6 (5.6)**	**3 (5.4)**	1.000 ^a^
Essential tremor	1 (16.7)	3 (100)	
Space occupying lesion	1 (16.7)	0 (0.0)	
Lumbar spine disease/neuropathy	3	0 (0.0)	
Functional	1 (16.7)	0 (0.0)	
Other	**6 (5.6)**	**4 (7.1)**	0.736 ^a^
Falls (cause unspecified)	5 (83.3)	0 (0.0)	
Postural hypotension	1 (16.7)	0 (0.0)	
Osteoarthritis	0 (0.0)	1 (25.0)	
Soft tissue pathology	0 (0.0)	1 (25.0)	
Multifactorial	0 (0.0)	1 (25.0)	
Tremor secondary to valproate	0 (0.0)	1 (25.0)	

Bold values in column which are statistically significant

Values are number (percentage)

*PD* Parkinson’s disease; *VP* vascular parkinsonism; *DLB* dementia with Lewy bodies; *TIA* transient ischaemic attack; *FTD* fronto-temporal dementia; *AD* Alzheimer’s disease

^*^Chi-square test

^a^Fisher’s exact test

The median time from index symptom to movement disorder review was also significantly longer in PSP/CBD (1.96 years [IQR 1.07, 4.30]) compared to PD (0.57 years [IQR 0.11, 1.84] (*p* = 0.002). At their first movement disorder specialist review, 16 (53.3%) PSP/CBD patients received a specific diagnosis of PSP/CBD, compared to 22 (73.3%) PD patients (*p* = 0.18). Of these PSP/CBD patients diagnosed at their first movement disorder review, over a median follow-up of 3.2 (SD 2.6) years, the diagnosis remained unchanged in 14 (87.5%) (*n* = 13 PSP, *n* = 1 CBD) individuals. By their final movement disorder review, 23 (76.7%) PSP/CBD cases received their diagnosis compared to 100% of PD cases (*p* = 0.010). 7 (23.3%) PSP/CBD cases were only diagnosed after death, either by post-mortem (*n* = 5) or full case note review (*n* = 2). In four cases diagnosed with PSP at post-mortem only, their (ultimately incorrect) initial clinical diagnoses (PD *n* = 2, VP *n* = 1 and MSA *n* = 1) remained unchanged over clinical follow-up.

## Discussion

Time intervals to key diagnostic milestones were significantly longer in PSP/CBD compared to age- and sex-matched PD at each successive stage of the evolving diagnostic process from index symptom onset. In addition to the greater delay in identifying parkinsonism (which influences treatments and narrows differential diagnoses), it is notable that PSP/CBD appears not to be readily considered, even once the parkinsonian syndrome has been identified. The complexity of the diagnostic journey for individuals with PSP/CBD was also greater than for those with PD, with more differential diagnoses, primary diagnoses and changes to diagnosis overall. Individuals with PSP/CBD had more emergency admissions prior to diagnosis and a greater number of specialties involved in their diagnostic process with longer delays to a movement disorder specialist review, in particular, which was a key step in making a diagnosis.

Some previous studies in PSP/CBD report similar time delays [10, 11], some longer [12], and some shorter [5, 13]. None, however, are directly comparable due to differences in the precision and method of symptom onset definition, for example self-report versus first documentation in the medical record, or unclear definitions of symptom onset. In PD, the time to diagnosis is often rapid (9–15 months) [14–16], consistent with our study findings, though longer time intervals are reported [17–19]. Extending comparisons to other neurodegenerative diseases, the time intervals from symptom onset to diagnosis in PSP/CBD are also longer than those reported for MND [20–29], which range from 9.3 [20] to 16.2 months [29], and are most similar to diagnostic intervals reported in dementia, especially frontotemporal dementia [30–33]. Fewer studies have evaluated the frequency of changes to diagnosis in PSP and CBD. Of 4141 cognitively impaired patients visiting a dementia research centre approximately a year apart, 19 PSP patients received their diagnosis on both visits, four on their first visit only and eight their second visit only. In CBD, 45 received the diagnosis on both visits, 14 the first visit only, and 15 the second visit only [34].

In general, there has been little research into the determinants of diagnostic delay in PSP/CBD. In one previous study, there was a significant correlation between diagnostic latency and age of onset, with older patients having shorter diagnostic latency overall (*r* =  − 0.23, *p* < 0.001). The average age of our cohort was 78 years and so the time intervals reported may therefore be even worse in younger patients. In PD proposed determinants include sex (longer for men to present to primary care) [16], motor phenotype (longer in non-tremor dominant presentations) [16, 35], and greater motor and non-motor severity [35], though the association of greater disease severity with longer diagnostic delay would seem somewhat counterintuitive.

An initial appraisal of presenting symptoms/signs is a critical first step to systematically evaluate the greater diagnostic uncertainty and delay in PSP/CBD. The high prevalence of postural instability at symptom onset in PSP/CBD suggests it is underappreciated as a core feature of atypical parkinsonism. If realised earlier, this might narrow differential diagnoses and expedite movement disorder review. This is reflected in more recent changes to diagnostic criteria. Although postural instability, for example, is included in the UK PD Brain Bank criteria, it is not included in the 2015 Movement Disorder Society PD criteria as its presence in early disease should prompt consideration of alternative diagnoses, including PSP. Key identified categorical misdiagnoses, particularly vascular or primary psychiatric disorders, could be reduced by targeted history and examination. The higher frequency of TIA/stroke diagnoses in PSP/CBD (*p* = 0.012), for example, suggests that focal symptoms or signs in PSP/CBD are being identified but misattributed, perhaps due to a clinical reasoning process favouring common diseases. Careful assessment of the speed of symptom onset and symptom progression should broadly differentiate vascular and neurodegenerative pathologies. The greater number of neuropsychiatric diagnoses in PSP/CBD (*p* < 0.01), suggests training psychiatrists to perform targeted motor examinations in those with predominantly cognitive or behavioural presentations may reduce misdiagnosis in PSP/CBD or trigger movement disorder specialist referral, an approach previously suggested to differentiate DLB and AD [36]. Clinicians also need to be aware of the tendency to over-diagnose PD once parkinsonism is recognised, which was the case in over 50% of our PSP/CBD cases, similar to the extent of overdiagnosis in other studies [2, 37]. Atypical parkinsonian disorders were rarely included amongst differential diagnoses for identified parkinsonism, suggesting available red flags such as early falls are not being recognised. The high prevalence of tremor in PD may partially explain the shorter time to final diagnosis, being particularly amenable to clinician diagnostic pattern recognition. Tremor pattern recognition probably also shortens patient appraisal delay, removing potential barriers to presentation as patients may delay attending their GP with non-specific symptoms [38].

The transition from primary to secondary care is another key milestone in the diagnostic process. While there are no national referral recommendations for PSP/CBD in the UK, in PD national guidelines explicitly encourage urgent referral to movement disorder specialists to expedite diagnosis, determine prognosis, prevent distress arising from diagnostic delay, and allow early multidisciplinary involvement [39–41], a rationale which is clearly also applicable to PSP/CBD. PSP/CBD patients, however, take approximately 8 months from index symptom to be referred to secondary care, longer than those with PD. As a similar proportion (66.7%) of index symptoms in both PSP/CBD and PD are identified in primary care, the longer transition from primary to secondary care identified in PSP/CBD may, in part, reflect a greater reliance on “test of time” diagnostic strategies [42] to evaluate non-specific symptoms, as well as a greater difficulty in attributing non-specific symptoms/signs to PSP/CBD, or indeed parkinsonism. The differential diagnoses formulated to explain such symptoms at this stage are critical however as this influences the location, as well as timing, of subsequent referrals. No PSP/CBD secondary care referral, for example, queried a specific diagnosis of PSP/CBD, likely due to its rarity within primary care, in contrast to 63% of PD referrals, while parkinsonism was proposed in only a third of PSP/CBD patient referrals compared to 70% in the PD group. The greater tendency for GPs in PSP/CBD to misattribute identified symptoms to non-parkinsonian conditions is also likely an important determinant of subsequent diagnostic delay as this frequently resulted in a breadth of referrals to medical and surgical specialities. Our findings are in keeping with a study of referral patterns in pathologically confirmed cases of PSP and PD from the Queen Square Brain Bank [37]. The Step-Back PSP study, while primarily evaluating symptoms in the pre-diagnostic period (the period at least 1 year prior to diagnosis), also noted that PSP patients had more consultations to ENT (*p* = 0.028) and ophthalmology (*p* = 0.016) compared to age- and sex-matched PD [12].

For nearly a third of PSP/CBD patients, predominantly women, their first secondary care contact after symptom onset is not due to a GP referral but an emergency presentation, three times higher than in their age-sex matched PD counterparts. Given the high fracture rate in those presenting with falls, the reported female preponderance in this group may reflect a higher fracture risk due to lower bone mineral density in women. Initial emergency presentations in PSP/CBD, unlike PD, also do not reliably trigger subsequent outpatient referrals to relevant specialities, resulting in a higher frequency of recurrent emergency attendances prior to outpatient referral and review. Such emergency attendances (predominantly due to falls) result in a high rate of hospitalisation (over 90% of all attendances in both groups). The longer duration of hospitalisation in those with PSP/CBD compared to PD, suggests that the rehabilitation of PSP/CBD patients, prior to diagnosis, may already be slower than their age-sex matched PD counterparts. In addition, unscheduled admissions rarely resulted in inpatient diagnoses, a missed diagnostic opportunity. While patients with atypical parkinsonism have been previously identified as having a higher fracture risk in the 2 years preceding diagnosis [43], the current analysis suggests that, in addition to falls and fractures, recurrent emergency presentations associated with a lengthy rehabilitative process, may serve as additional diagnostic red flags for PSP/CBD.

PSP/CBD is also difficult to diagnose even once individuals have reached secondary care unless there is movement disorder expertise. PSP/CBD was diagnosed in only 11.1% of initial outpatient reviews in the PSP/CBD group compared to 60.0% diagnosed with PD in the PD group, while parkinsonism was diagnosed in 40% of PSP/CBD cases and two-thirds of PD cases after an initial outpatient neurology or COTE review. Although PSP/CBD patients first reached secondary care by approximately 8 months on average, it took individuals with PSP/CBD nearly 2 years from symptom onset to receive specialist movement disorder input, approximately three times longer than PD patients. This is shorter than the time reported in a series of 16 pathologically confirmed PSP cases where the time to first movement disorder clinic visit was 2.9 years [11]. The necessity of such input, particularly in PSP/CBD, is evident in that only one individual received a diagnosis of PSP/CBD prior to movement disorder review, in contrast to 24 (80.0%) PD cases who received their diagnosis prior to this input. In PD in contrast a previous review has shown that the accuracy of diagnosis in PD is not significantly lower in non-experts (73.8%) [44]. In addition, in a more recent retrospective survey of 1775 patients with PD, there was no difference in diagnostic certainty between a general neurologist or movement disorder neurologist [45].

Having reached a movement disorder specialist, approximately 50% of those with PSP/CBD were diagnosed at their first movement disorder assessment, reaching approximately 75% by their final review. Several studies have previously highlighted the improved diagnostic accuracy at final compared to initial review [2, 5, 37]. Whilst movement disorder specialists may be particularly adept at pattern recognition and have a greater familiarity with atypical features or rarer phenotypes [46], 25% of PSP/CBD did not receive their diagnosis in life, serving to highlight the persistent difficulties in diagnosis, even after movement disorder specialist input.

Unexpectedly, a novel finding was that there was little difference in the overall survival from carefully documented symptom onset to death in those with PSP/CBD and their age- and sex- matched PD counterparts. In PD, older age and male sex have been identified as independent worse prognostic factors. Due to 1:1 sex-matching in PD to match the PSP/CBS sex ratio, survival in PD in our sample has likely been extended as relatively more women were included in the PD group. However, conversely, in that the median age in PSP (79.9 years) was also older than the entire incident PD population in PINE (73.8 years), age matching may have decreased survival in our matched PD population. At the time of the study, the NINDS-PSP criteria were used for diagnosis which did not permit the diagnosis of non-Richardson’s syndrome subtypes of PSP. Given the PINE study was an incident cohort of parkinsonism, cortical PSP subtypes (which may have greater diagnostic delay and a poorer prognosis) are likely under-represented potentially extending survival in our PSP cohort. However, the proportion of cognitive subtypes are relatively small [47, 48] and unless cases had a purely cognitive presentation (therefore likely excluding the corticobasal presentations included within the cognitive subtype), they are likely to have been identified within the PINE study. Finally, unlike the lead-time bias seen in association with screening programmes, whereby an earlier diagnosis due to screening makes it appear as if individuals are surviving longer, the contribution of diagnostic delay should also be considered. Ultimately our findings relating to survival from index symptom require replication in a bigger incidence cohort (requiring multi-centre involvement), with access to primary care records to identify index symptoms.

A particular strength of this study is the inclusion of the entire recruited PSP/CBD population of a community based, prospective incident study of parkinsonism in the UK, meaning PSP/CBD cases are likely to be representative of the motor predominant PSP/CBD population. Additional strengths include the detailed systematic review of initial symptoms and signs from primary and secondary care records, the availability of detailed longitudinal data from research and clinical records, and the comparative analysis in PSP/CBD and PD matching on variables identified as possible determinants of diagnostic delay.

The study is not without limitations. PSP and CBD are distinct diseases with differentiating clinical features but were considered together for analysis due to small numbers (but akin to many studies in PSP/CBD due to their rarity). There is significant overlap between PSP and CBD clinically, but nonetheless with two CBD cases, our results mostly apply to those with PSP. The PINE study is an incidence study of parkinsonism so, while representative of the motor predominant PSP/CBD population, may under-represent those with a predominantly cognitive initial presentation. Primary and secondary care referrals of patients to a neurologist with a specialist interest in movement disorders were also actively sought as part of the PINE study, which may have reduced the time to movement disorder specialist and increased diagnostic accuracy compared to real-world practice in the UK. Unless explicitly documented, it is impossible to determine how frequently differential diagnoses or changes to primary diagnosis were communicated to patients, and the extent to which this impacted their perception and experience of their diagnostic journey. Pathological verification of all clinical diagnoses was not achieved (although our 30% post-mortem confirmation is good for a clinical study). This is never possible outside brain bank studies which, as detailed previously, are highly selected and susceptible to bias. During life clinicians rely on clinical diagnosis to guide management, and so even without pathological confirmation of all cases, studies on best clinical diagnosis are important. In addition, final clinical diagnostic accuracy in our study is also likely to be high due to, at minimum, annual reviews from diagnosis to death and a full case note review after death. While, without post-mortem data in all cases, we may have missed some with atypical PSP, the effect of this potential bias would tend to make “true” delays in all cases of PSP/CBD even longer without better diagnostic tests. The PINE study also preceded the updated MDS diagnostic criteria for PSP [49]. While this is unlikely to have altered diagnosis in most cases and there is some evidence that clinical expert diagnosis is better than rigid research criteria [50], it has prevented subtyping of PSP cases in our cohort which may have masked potential differences in diagnostic delay and survival between subtypes. Implementation of relatively complex diagnostic criteria however takes time and so our study will likely reflect the day-to-day clinical diagnostic process and experience of PSP/CBD patients in the UK.

In conclusion, individuals with PSP/CBD had a longer and more complex diagnostic pathway than individuals with age-sex matched PD. There are several ways this could be improved including greater recognition that balance issues may be parkinsonian, raised awareness of red flags for PSP including emergency admissions preceding diagnosis for unexplained falls/fractures and earlier access to movement disorder specialists. We found little difference in overall survival from symptom onset to death in PSP/CBD and age-sex matched PD. If replicated, this has important implications for our understanding of prognosis in PSP/CBD.

## Supplementary Information

Below is the link to the electronic supplementary material.

Supplementary file1 (DOCX 26 KB)

## References

[1] Josephs KA, Dickson DW (2003). Diagnostic accuracy of progressive supranuclear palsy in the society for progressive supranuclear palsy brain bank. Mov Disord.

[2] Osaki Y, Ben-Shlomo Y, Lees AJ (2004). Accuracy of clinical diagnosis of progressive supranuclear palsy. Mov Disord.

[3] Ling H, O’Sullivan SS, Holton JL (2010). Does corticobasal degeneration exist? A clinicopathological re-evaluation. Brain.

[4] Litvan I, Agid Y, Goetz C (1997). Accuracy of the clinical diagnosis of corticobasal degeneration: a clinicopathologic study. Neurology.

[5] Respondek G, Roeber S, Kretzschmar H (2013). Accuracy of the national institute for neurological disorders and stroke/society for progressive supranuclear palsy and neuroprotection and natural history in Parkinson plus syndromes criteria for the diagnosis of progressive supranuclear palsy. Mov Disord.

[6] Fielding S, Macleod AD, Counsell CE (2016). Medium-term prognosis of an incident cohort of parkinsonian patients compared to controls. Parkinsonism Relat Disord.

[7] Litvan I, Agid Y, Calne D (1996). Clinical research criteria for the diagnosis of progressive supranuclear palsy (Steele–Richardson–Olszewski syndrome): report of the NINDS-SPSP international workshop. Neurology.

[8] Lang AE, Riley DE, Bergeron C, Calne DB (1994). Cortical-basal ganglionic degeneration. Neurodegenerative diseases.

[9] Hughes AJ, Daniel SE, Kilford L, Lees AJ (1992). Accuracy of clinical diagnosis of idiopathic Parkinson’s disease: a clinico-pathological study of 100 cases. J Neurol Neurosurg Psychiatry.

[10] Coyle-Gilchrist IT, Dick KM, Patterson K (2016). Prevalence, characteristics, and survival of frontotemporal lobar degeneration syndromes. Neurology.

[11] Birdi S, Rajput AH, Fenton M (2002). Progressive supranuclear palsy diagnosis and confounding features: report on 16 autopsied cases. Mov Disord.

[12] Painous C, Martí MJ, Simonet C (2020). Prediagnostic motor and non-motor symptoms in progressive supranuclear palsy: the step-back PSP study. Parkinsonism Relat Disord.

[13] Mamarabadi M, Razjouyan H, Golbe LI (2018). Is the latency from progressive supranuclear palsy onset to diagnosis improving?. Mov Disord Clin Pract.

[14] Rana AQ, Siddiqui I, Yousuf MS (2012). Challenges in diagnosis of young onset Parkinson’s disease. J Neurol Sci.

[15] Saunders-Pullman R, Wang C, Stanley K, Bressman SB (2011). Diagnosis and referral delay in women with Parkinson’s disease. Gend Med.

[16] Breen DP, Evans JR, Farrell K, Brayne C, Barker RA (2013). Determinants of delayed diagnosis in Parkinson’s disease. J Neurol.

[17] Femi OL, Ibrahim A, Aliyu S (2012). Clinical profile of parkinsonian disorders in the tropics: experience at Kano, northwestern Nigeria. J Neurosci Rural Pract.

[18] Okubadejo NU, Ojo OO, Oshinaike OO (2010). Clinical profile of parkinsonism and Parkinson’s disease in Lagos, Southwestern Nigeria. BMC Neurol.

[19] Lay-Son L, Eloiza C, Trujillo-Godoy O (2015). Delay in the diagnosis of Parkinson’s disease in a Chilean public hospital. Rev Med Chil.

[20] Zoccolella S, Beghi E, Palagano G (2006). Predictors of delay in the diagnosis and clinical trial entry of amyotrophic lateral sclerosis patients: a population-based study. J Neurol Sci.

[21] Nzwalo H, de Abreu D, Swash M, Pinto S, de Carvalho M (2014). Delayed diagnosis in ALS: the problem continues. J Neurol Sci.

[22] Turner MR, Scaber J, Goodfellow JA, Lord ME, Marsden R, Talbot K (2010). The diagnostic pathway and prognosis in bulbar-onset amyotrophic lateral sclerosis. J Neurol Sci.

[23] Rocchetti I, Taruscio D, Pierannunzio D (2012). Modeling delay to diagnosis for amyotrophic lateral sclerosis: under reporting and incidence estimates. BMC Neurol.

[24] Cellura E, Spataro R, Taiello AC, La Bella V (2012). Factors affecting the diagnostic delay in amyotrophic lateral sclerosis. Clin Neurol Neurosurg.

[25] Galvin JE, Duda JE, Kaufer DI, Lippa CF, Taylor A, Zarit SH (2010). Lewy body dementia: the caregiver experience of clinical care. Parkinsonism Relat Disord.

[26] Kraemer M, Buerger M, Berlit P (2010). Diagnostic problems and delay of diagnosis in amyotrophic lateral sclerosis. Clin Neurol Neurosurg.

[27] Chiò A (1999) ISIS survey: an international study on the diagnostic process and its implications in amyotrophic lateral sclerosis. J Neurol 246(Suppl 3): III1–III5 10.1007/BF03161081 10631652

[28] Donaghy C, Dick A, Hardiman O, Patterson V (2008). Timeliness of diagnosis in motor neurone disease: a population-based study. Ulster Med J.

[29] Househam E, Swash M (2000). Diagnostic delay in amyotrophic lateral sclerosis: what scope for improvement?. J Neurol Sci.

[30] Boise L, Morgan DL, Kaye J, Camicioli R (1999). Delays in the diagnosis of dementia: perspectives of family caregivers. Am J Alzheimers Dis.

[31] Speechly CM, Bridges-Webb C, Passmore E (2008). The pathway to dementia diagnosis. Med J Aust.

[32] Draper B, Cations M, White F (2016). Time to diagnosis in young-onset dementia and its determinants: the INSPIRED study. Int J Geriatr Psychiatry.

[33] Zhao M, Lv X, Tuerxun M (2016). Delayed help seeking behavior in dementia care: preliminary findings from the clinical pathway for Alzheimer’s Disease in China (CPAD) study. Int Psychogeriatr.

[34] Koepsell TD, Gill DP, Chen B (2013). Stability of clinical etiologic diagnosis in dementia and mild cognitive impairment: results from a multicenter longitudinal database. Am J Alzheimers Dis Other Demen.

[35] Wan Y, Zhu Y, Luo Y (2019). Determinants of diagnostic latency in Chinese people with Parkinson’s disease. BMC Neurol.

[36] Thomas AJ, Taylor JP, McKeith I (2018). Revision of assessment toolkits for improving the diagnosis of Lewy body dementia: the DIAMOND Lewy study. Int J Geriatr Psychiatry.

[37] Williams DR, Lees AJ (2009). How do patients with parkinsonism present? A clinicopathological study. Intern Med J.

[38] Plouvier AO, Olde Hartman TC, Boots LP, Bloem BR, van Weel C, Lagro-Janssen AL (2015). Time intervals in diagnosing Parkinson’s disease: the patients’ views. Patient Educ Couns.

[39] Scottish Intercollegiate Guidelines Network (SIGN) (2010) Diagnosis and pharmacological management of Parkinson’s disease: a national clinical guideline (SIGN Guideline No 113)

[40] National Institute for Health and Care Excellence (NICE) (2017) NICE guideline [NG71]: Parkinson’s disease in adults

[41] European Parkinson’s Disease Association (EPDA) (2011) The European Parkinson’s disease standards of care consensus statement

[42] Heneghan C, Glasziou P, Thompson M (2009). Diagnostic strategies used in primary care. BMJ.

[43] Sleeman I, Che ZC, Counsell C (2016). Risk of fracture amongst patients with Parkinson’s disease and other forms of parkinsonism. Parkinsonism Relat Disord.

[44] Rizzo G, Copetti M, Arcuti S, Martino D, Fontana A, Logroscino G (2016). Accuracy of clinical diagnosis of Parkinson disease: a systematic review and meta-analysis. Neurology.

[45] Schrag A, Khan K, Hotham S, Merritt R, Rascol O, Graham L (2018). Experience of care for Parkinson’s disease in European countries: a survey by the European Parkinson’s Disease Association. Eur J Neurol.

[46] Hughes AJ, Daniel SE, Ben-Shlomo Y, Lees AJ (2002). The accuracy of diagnosis of parkinsonian syndromes in a specialist movement disorder service. Brain.

[47] Street D, Malpetti M, Rittman T (2021). Clinical progression of progressive supranuclear palsy: impact of trial bias and phenotype variants. Brain Commun.

[48] Jabbari E, Holland N, Chelban V (2020). Diagnosis across the spectrum of progressive supranuclear palsy and corticobasal syndrome. JAMA Neurol.

[49] Höglinger GU, Respondek G, Stamelou M (2017). Clinical diagnosis of progressive supranuclear palsy: the movement disorder society criteria. Mov Disord.

[50] Caslake R, Moore JN, Gordon JC, Harris CE, Counsell C (2008). Changes in diagnosis with follow-up in an incident cohort of patients with parkinsonism. J Neurol Neurosurg Psychiatry.

