# Supplementary material for: The evolution of diagnosis from symptom onset to death in progressive supranuclear palsy (PSP) and corticobasal degeneration (CBD) compared to Parkinson’s disease (PD)
Source: J Neurol. 2023 Mar 27;270(7):3464–74. doi: 10.1007/s00415-023-11629-x (PMC10266988; doi:10.1007/s00415-023-11629-x)
Supplement: Supplementary file 1 — Supplementary file1 (DOCX 26 KB) [file 415_2023_11629_MOESM1_ESM.docx]

**Appendix 1**

1. Index symptoms, namely:

balance difficulties, falls, slowness/immobility, freezing, stiffness, tremor, visual disturbance, speech or voice change, neuropsychiatric symptoms, cognitive symptoms, swallowing difficulties, weight loss, constipation, urinary symptoms, urinary or faecal incontinence, sleep disturbance, fatigue, pain, dystonic symptoms, sensory symptoms, difficulty with coordination);

2. Index signs, namely:

nystagmus, involuntary eye closure, diplopia, slowed vertical saccades, slow horizontal saccades, vertical gaze palsy, speech or voice change, rigidity, tremor, bradykinesia, weakness, sensory disturbance, abnormal reflexes, primitive reflexes, gait disturbance, impaired balance^*^, dyspraxia, dystonia, myoclonus and ^≠^cognitive impairment),

^*^Impaired balance was defined as evidence of postural instability on a pull test, ^ǂ^the presence of falls required an Accident and Emergency (A&E) attendance or hospital admission due to a fall, ^≠^MMSE of ≤28 was considered evidence of at least mild cognitive impairment.

**Supplementary Table 1** Comparison of index sign frequency (categorised) in PSP/CBD and PD

| **Index signs** | **PSP/CBD**  **n=30** | **PD**  **n=30** | **p-value*** |
| --- | --- | --- | --- |
| Tremor | 3 [3 PSP] (10.0%) | 21 (70.0%) | **<0.001** |
| Bradykinesia | 3 [3 PSP] (10.0%) | 9 (30.0%) | 0.053 |
| Gait disturbance | 11 [11 PSP] (36.7%) | 14 (46.7%) | 0.598 |
| Rigidity | 5 [5 PSP] (16.7%) | 13 (43.3%) | **0.024** |
| Impaired balance | 15 [14 PSP, 1 CBD] (50.0%) | 7 (23.3%) | **0.032** |
| Falls | 11 [11 PSP] (36.7%) | 2 (6.7%) | **0.005** |
| Slowed vertical saccades | 2 [2 PSP] (6.7%) | 0 (0.0%) | 0.150 |
| Vertical gaze palsy | 0 (0.0%) | 0 (0.0%) | - |
| Speech/voice | 6 [6 PSP] (20.0%) | 9 (30.0%) | 0.371 |
| Weakness | 2 [2 PSP] (6.7%) | 0 (0.0%) | 0.150 |
| Abnormal reflexes | 4 [3 PSP, 1CBD] (13.3%) | 0 (0.0%) | 0.038 |
| Primitive reflexes | 2 [2 PSP] (6.7%) | 0 (0.0%) | 0.150 |
| Sensory disturbance | 2 [1 PSP, 1 CBD] (6.7%) | 0 (0.0%) | 0.150 |
| Dystonia | 0 (0.0%) | 0 (0.0%) | - |
| Dyspraxia | 2 [1 PSP, 1 CBD] (6.7%) | 0 (0.0%) | 0.150 |
| Myoclonus | 1 [1 PSP] (3.3%) | 0 (0.0%) | 0.313 |
| Cortical sensory loss | 1 [1 PSP] (3.3%) | 0 (0.0%) | 0.313 |
| Cognitive impairment (MMSE ≤28) | 2 [1 PSP] (6.7%) | 0 (0.0%) | 0.150 |

PSP= progressive supranuclear palsy, CBD= corticobasal degeneration, PD = Parkinson’s disease *Chi-squared test

**Supplementary Table 2** Comparison of time intervals from patient recalled index symptom to diagnosis in PSP/CBD and age-sex matched PD

| **Median (IQR) years from patient recalled index symptom:** | **PSP/CBD**  **n=30** | **PD**  **n=30** | **p-value*** |  |
| --- | --- | --- | --- | --- |
| To diagnosis of a parkinsonian syndrome | 1.34  (0.70, 2.26) | 0.96  (0.37, 1.60) | **0.040** |  |
| To specific diagnosis in differential diagnoses | 2.36  (1.25, 4.00) | 1.02  (0.58, 1.61) | **0.001** |  |
| To specific diagnosis as primary clinical diagnosis (initial) | 2.82  (1.51, 4.23) | 1.02  (0.64, 2.00) | **<0.001** |  |
| To specific diagnosis as primary clinical diagnosis (unchanging) | 3.24  (2.01, 4.81) | 1.59  (0.97, 3.00) | **0.009** |  |
| To death | 4.96  (3.67, 7.80) | 6.45  (4.29, 9.47) | 0.156 |  |

**Supplementary Table 3** Comparison of time intervals from index sign to diagnosis in PSP/CBD and age-sex matched PD

| **Median (IQR) years from index sign:** | **PSP/CBD**  **n=30** | **PD**  **n=30** | **p-value*** |
| --- | --- | --- | --- |
| To diagnosis of a parkinsonian syndrome | 1.32  (0.19, 2.40) | 0.08  (0.00, 0.75) | **0.002** |
| To specific diagnosis in differential diagnoses | 2.48  (0.98, 4.02) | 0.18  (0.00, 1.48) | **<0.001** |
| To specific diagnosis as primary clinical diagnosis (initial) | 3.21  (1.47, 5.10) | 0.18  (0.04, 1.48) | **<0.001** |
| To specific diagnosis as primary clinical diagnosis (unchanging) | 3.30  (1.84, 5.46) | 0.70  (0.06, 3.26) | **0.001** |
| To death | 5.18  (3.17, 7.36) | 6.37  (3.33, 8.59) | 0.249 |

PSP= progressive supranuclear palsy, CBD= corticobasal degeneration, PD = Parkinson’s disease. *Mann-Whitney

**Supplementary Table 4** Reason and duration of admission in PSP/CBD patients with emergency attendances and hospital admissions prior to outpatient referral or inpatient movement disorder review

| Reason for emergency attendance | Emergency attendances prior to outpatient referral  n=27 | Hospitalisation prior to outpatient referral  n=24 | Duration of admission (days) |
| --- | --- | --- | --- |
| Falls  No significant injury  Fracture  Neck of femur  Radius  Facial  Head injury  Other injury | 15 (55.6%)  5 (33.3%)  5 (33.3)  3 (60.0)  1 (20.0)  1 (20.0)  3 (20.0)  2 (13.3) | 12 (50.0)  3 (25.0)  5 (41.7)  3 (60.0)  1 (20.0)  1 (20.0)  3 (25.0)  1 (8.3) | 21.0 (11.8, 45.8)  24.0 (22.5, 63.5)  43.0 (15.0, 54.0)  54.0 (34.5, 58.5)  43.0  2.0  2.0 (2.0, 9.5)  21.0 |
| Reduced mobility | 4 (14.8) | 4 (16.7) | 30.5 (15.5, 46.3) |
| Neuropsychiatric | 5 (18.5) | 5 (17.2) | 61.0 (42.0, 333.0) |
| Other | 3 (11.1) | 3 (12.5) | 9.0 (5.8, 13.3) |
| Total | 27 (100) | 24 (100) | 21.0 (14.0, 53.3) |

Values are frequency (percentage) and median (interquartile range)
